# CMR Assessment of the Left Ventricle Apical Morphology in Subjects with Giant T-wave Inversions and Without Apical Wall Thickness>15mm

**DOI:** 10.1186/1532-429X-18-S1-P277

**Published:** 2016-01-27

**Authors:** Minjie Lu, Bailing Wu, Yan Zhang, Peter Kellman, Mehul B Patel, Jongmin Lee, Shihua Zhao

**Affiliations:** 1 grid.415105.4Magnetic Resonance Imaging, Fuwai Hospital, Beijing, China; 2 grid.452702.60000000418043009Radiology, the Second Hospital of Hebei Medical University, Shijiazhuang, China; 3 Cardiovascular and Pulmonary Branch, National Heart, Lung and Blood Institute, National Institutes of Health, US Department of Health and Human Services, Bethesda, MD USA; 4 grid.39382.33000000012160926XBaylor College of Medicine, Houston, TX USA; 5 grid.258803.40000000106611556Radiology, Biomedical Engineering and Medical Science, Kyungpook National University, School of Medicine, Daegu, Korea (the Democratic People's Republic of)

## Background

Unexplainable giant T-wave inversions in the precordial leads with apical wall thickness <15 mm has been reported. These patients cannot be diagnosed to have apical hypertrophic cardiomyopathy (AHCM) according to the current criteria. The objective of this study was to evaluate the apical morphology of subjects with giant T-wave inversion in the absence of apical wall thickness>15 mm using cardiac magnetic resonance (CMR).

## Methods

A total of 60 subjects with giant T-wave inversions and 76 healthy volunteers were enrolled in the study. The segmented left ventricular (LV) wall thickness was measured according the American Heart Association 17-segmented model. The apical angle (apA) as well as the ratios of the segmented thickness to that of basal posterior wall was calculated. The regional variations of LV wall thickness were analyzed among basal, middle and apical walls as well as septum and free wall.

## Results

Considerable variation in LV wall thickness in normal subjects was observed across the ventricle with progressive thinning from the base to apex (male and female p < 0.001). The apical thickness of subjects with T wave inversions was 8.10 ± 1.67 mm in male, which is thicker than that of controls (4.14 ± 1.17 mm, p < 0.001). In female the apical thickness also had significant difference as controls (5.85 ± 2.16 mm versus 2.99 ± 0.65 mm p = 0.007). Compared with normals, the apical angle decreased significantly in male (87.44 ± 13.86° vs. 115.03 ± 9.90, p < 0.001) and female (90.69 ± 8.84° vs. 110.07 ± 13.58°, p < 0.001) subjects, respectively.

## Conclusions

The apical morphology of subjects with giant T-wave inversion is significantly different from normal, although the absolute thickness of the LV apical wall is below current diagnostic criteria of AHCM. We may propose that it is the apical angle/or the thickness that determines the genesis of precordial T wave inversion. Further investigations are needed to testify whether these subjects should be included within the scope of AHCM or whether this represents a benign new variant.

